# Experiences of labor-intensive procedures in the management of nocturnal enuresis—an explorative interview study in children and parents

**DOI:** 10.1371/journal.pone.0314623

**Published:** 2024-12-02

**Authors:** Malin Borgström, Katarina Hjelm, Barbro H. Skogman, Tryggve Nevéus, Maria Tunebjer

**Affiliations:** 1 Department of Women’s and Children’s Health, Uppsala University, Uppsala, Sweden; 2 Center for Clinical Research Dalarna, Uppsala University, Falun, Sweden; 3 Department of Public Health and Caring Science, Uppsala University, Uppsala, Sweden; 4 Örebro University, Örebro, Sweden; SickKids: The Hospital for Sick Children, CANADA

## Abstract

**Introduction:**

Nocturnal enuresis is a very common and socially distressing condition among children. There are qualitative studies published in children with bowel and bladder problems with a focus on health-related quality of life and the burden of the condition, but there is a lack of knowledge of the experience of managing the treatment procedures at home. From a clinical viewpoint, it can be assumed that the procedures entail a lot of work and have an impact on everyday life apart from the disorders per se, but the actual voices of the children have not been heard.

**Purpose:**

To explore children’s and their parents’ experiences of labor-intensive procedures in the management of enuresis.

**Design and methods:**

A qualitative interview study. Semi-structured interviews were held with fourteen children with enuresis six to nine years of age, together with their parents in Sweden between April 2020 and February 2021. Data were analyzed inductively with qualitative content analysis.

**Results:**

The major findings showed both favorable strategies and challenges in labor-intensive management. The results are described in four categories: 1) experiences of how to manage; 2) managing procedures was a favorable experience; 3) managing procedures made it complicated and 4) problematic to remember.

**Conclusion:**

Managing the treatment of enuresis at home can be challenging. There were procedures that children did not like and new routines that were difficult to remember. However, the study can suggest strategies in how to manage and overcome these difficulties.

## Introduction

Nocturnal Enuresis is a common condition; approximately 5–10% of seven year-olds [1] and 3–5% of ten year-olds are affected [2]. Prevalence figures and developmental trajectories are similar between countries and cultures [1]. Enuresis is troublesome for the whole family and contributes to isolation due to fear of wet nights during sleepovers and school trips [3]. There is no treatment for enuresis that fits all, which means that the time between start of treatment and dryness can be long [4].

Studies on children’s and parents’ experiences of enuresis confirm a considerable detrimental effect on quality of life [5–7]. However, there are so far no studies exploring children’s and parents’ experiences of the management of enuresis. The United Nations Convention on the Rights of the Child, which has been elevated to law in Sweden since 2018, emphasizes the child’s right to express their own opinion [8] as well as the need to consider the child’s perspective in decision-making. A nurse or physician can evaluate enuresis and initiate treatment, as long she or he has the knowledge and competence to do so [4], but the child’s opinion always needs to be taken into account. This means that we need to know more about the children’s experiences of the various therapies, especially those that are labor-intensive or that intrude on the child’s integrity.

Three treatment modalities were, according to international guidelines, recommended as first-line therapies in enuresis at the time of the study implementation: 1) standard urotherapy, 2) alarm treatment, and 3) desmopressin [9]. Standard urotherapy is a form of non-pharmacological, non-surgical bladder rehabilitation based on behavioral modifications. However, recent studies have questioned the efficacy of standard urotherapy for children with enuresis without daytime incontinence [10–12].The enuresis alarm has long been established as a first-line antienuretic treatment [13].

Among children with daytime incontinence [14] and probably those with enuresis [15], constipation is a common condition [16], and is recommended to be eradicated before start of active treatment of the enuresis per se [17]. Standard urotherapy, alarm treatment and constipation treatment are all labor-intensive therapies, whereas desmopressin is much less disruptive, just a pill given at bedtime [18].

According to international guidelines [19], the voiding chart and bladder/bowel diary are crucial tools in the initial evaluation of children with enuresis, since it is assumed that they add information regarding pathogenesis and optimal treatment.

There are qualitative studies published about children with bowel and bladder problems with a focus on health-related quality of life and the burden of the conditions [2, 6], but there is a lack of knowledge of the experience of managing the treatment procedures at home. From a clinical viewpoint, it can be assumed that the procedures entail a lot of work and have an impact on everyday life apart from the disorders per se, but the actual voices of the children have not been heard. Knowledge gained from this investigation can empower children and parents, giving them an increased influence over the management of enuresis, while highlighting the treatment challenges and success factors. The aim of the study is to explore children’s and their parents’ experiences of labor-intensive procedures in the management of enuresis.

## Material and methods

### Study design

A qualitative explorative study design [20] was used to acquire in-depth knowledge in an area that has previously scarcely been studied. To get a deeper understanding of children’s and parents’ experiences, interviews with semi-structured questions were used to facilitate expression of thoughts and experiences, focusing on the given topic [20, 21].

### Study setting and recruitment

Children aged 6–9 years with primary monosymptomatic enuresis and experience of standard urotherapy, enuresis alarm and/or constipation treatment were recruited 9 April 2020–11 February 2021in Dalarna County, western central Sweden. The children were previous participants in other studies [11, 22] and were invited to participate in this interview study as well. Inclusion criteria to the former studies were children aged 6–11 years with enuresis. Exclusion criteria: daytime incontinence, previous urotherapy, previous treatment with the enuresis alarm or second-line antienuretic therapy, voiding dysfunction or suspected LUT malformation, concomitant disorders influencing renal or LUT function, inability to give informed consent or comply with instructions given. All treatments given during the research project were the same as if the children had been treated clinically. None of the children had a neuropsychiatric diagnosis at the time of the study.

The sample of children was selected purposively to ensure variation [20] since the intention was to include children of mixed genders and ages as well as varied experience of managing the included procedures. Since enuresis is more common in 6–7 year old children, a majority of the interviewed children were in that age. The interviewed children’s characteristics and experiences of procedures is presented in Table 1. The children were invited together with a parent. Before the interview, it was clarified that the child was the main character of the interview and the invitation of a parent was aimed at providing a context to the child’s story.

**Table 1:** Characteristics of the study participants, N = 14.

Participants together with a parent (n)	
Boys	9
Girls	5
Age of child, years (n)	
6	4
7	6
8	1
9	3
Experiences of procedures* (n)	
Voiding chart and bladder/bowel diary	14
Constipation treatment	7
Standard urotherapy	8
Alarm treatment	5
Outcome of enuresis treatment (n)	
Completely dry at night	1
Partial response, ≥ 50% reduction of wet nights	4
Nonresponse, less than 50% reduction of wet nights	9

*several study participants had experienced more than one procedure

A pediatric nurse/urotherapist/PhD candidate (MB) interviewed the children together with a parent, in a child-friendly room at a pediatric outpatient ward familiar to the child. The children could choose whether they wanted to sit in a chair or on a rocking toy, or move freely in the room.

### Data collection

The researchers formed the interview guide with semi-structured, open-ended questions based on professional experience of urinary tract problems, pediatrics and talking to children, in order to ensure that they were age-appropriate and could be understood by the children. The interview protocol was short and focused on the child’s description of the experience of performing the procedures related to relevant objects, as used at home. The objects related to the labor-intensive procedures; voiding chart and bladder/bowel diary, constipation treatment, standard urotherapy and alarm treatment are presented in Table 2. The children were asked to pick up the object they wanted to talk about one by one. In this way, the child was the one who chose the order in which the conversation would be conducted by describing and telling about his/her experience. Supplementary questions were asked to help the child to elaborate about the experiences. The method of using objects (props) is inspired from other research fields such as criminology where it is important and more common to interview younger children. Children can recall and provide richer answers if props are used to remind them of what to talk about [23, 24].

**Table 2:** Description of objects included in the labor-intensive procedures. The objects are used as props during the interviews.

Procedure	Objects/props
Voiding chart and bladder/bowel diary	Measure-cup and paper lists for registration
Constipation treatment	Mini-enema and bulk laxative, PEG 4000
Standard urotherapy	Toilet diary with stickers, water bottle, foot-stool
Alarm treatment	Alarm device, sensor and fixation in underwear (sanitary pads)

When the child brought up irrelevant subjects, the interviewer followed the child and mildly led the interview back to focus. During the interview the parent had an opportunity to add experiences based on their perspective.

A pilot study was conducted and revealed that it was difficult for the children to recall and describe their experiences even though the interviews were held no later than two weeks after end of treatment. The first interviews were held online due to pandemic restrictions and it then turned out that the children had difficulty focusing. Consequently, the interview technique was revised so that it was performed in person and the actual objects that were used at home were presented, as described above. This resulted in richer statements. Still, the interviews in the pilot study turned out to provide information fit to be included in the analysis. The interviews continued until an overall picture emerged and no new information was acquired [20].

### Data analysis

Data were analysed inductively with qualitative content analysis [20] and the intention was to adhere closely to the interviewee’s own words. Since children’s experiences of enuresis management at home was previously not explored, it was regarded as very important to describe their views and not to interpret what was said. All interviews were audio recorded and professionally transcribed verbatim. In summary, the interviews produced a transcript material of 268 A4 pages in font size 12 with double line spacing. The transcripts were read through to ensure that the material corresponded to the tape-recorded interviews. By reading the text several times a sense of the content as a whole was obtained. Meaning-bearing units were identified and coded close to the text. Codes with similar patterns were grouped together, resulting in subcategories, and finally sorted into categories, exemplified in Table 3.

**Table 3:** Example of categorization.

Category	Sub-category	Meaning unit
Managing procedures made it complicated	Awkward situations	*“I started laughing at myself and started joking about it and then*, *well*, *I was kind of afraid that my friends would notice that I had a box of sanitary pads in my room*.*"*

### Rigor and reflexivity

The interviewer (first author) is a PhD candidate and registered nurse with a specialist competence in pediatric nursing and 20 years of experience of communicating with children. She is also an urotherapist with special expertise of children in different ages with bladder and bowel problems.

Two of the authors (MB and MT, researchers in pediatric nursing), worked separately with coding and thereafter the codes were compared and discussed including a third author (KH, general nurse, researcher with vast experience in qualitative research), showing high similarity. The whole research group–with competence in qualitative research as well as pediatric nursing and medical care of children with bladder and bowel problems–was included in the final process of sorting codes into categories and subcategories until consensus was reached [20]. To further enhance trustworthiness of the findings, a thorough description of the decision trail and the procedure has been given. In order to help the reader judge transferability the characteristics of the study population and setting is provided. Confirmability was achieved by demonstrating categories with illuminative quotes from the participants’ responses.

### Ethical considerations

The study was conducted in accordance with the principles of the Declaration of Helsinki and was reviewed and approved by the Swedish Ethical Review Authority (dnr 2018/004, 2019–04670). Before inclusion, written informed consent was obtained from children and parents. Children were compensated with a ticket to a Playland. Data has been handled according to European General Data Protection Regulation Services (GDPR).

## Results

### Characteristics of the sample

In total, 14 child-parent pairs were interviewed. The individual interviews lasted 30–40 minutes. The children tended to share their experiences in short statements, while the answers from parents were more elaborate. The experiences of children and parents revealed success factors and challenges in labor-intensive management at home, due to enuresis and constipation. The result is described in four categories with subsequent subcategories, summarized in Table 4: 1) experiences of how to manage; 2) managing procedures was a favorable experience; 3) managing procedures made it complicated; 4) problematic to remember.

**Table 4:** Overview of the result.

Category (No. of statements)	Sub-category (No. of statements)
Experiences of how to manage (105)	This could be done in a better way (32)
	To be committed (29)
	Finding a way to remember (18)
	The child’s strategies (9)
	Solving problems (9)
	Distractors during enema administration (8)
Managing procedures was a favorable experience (100)	The urine measurement was interesting and easily to register (32)
	Standard urotherapy was easy to manage (25)
	Favorable experience of constipation treatment (17)
	Gratitude for increased knowledge (15)
	Alarm treatment being satisfying (11)
Managing procedures made it complicated (99)	Complications related to alarm treatment (40)
	Standard urotherapy was difficult to manage (23)
	Constipation treatment was troublesome (19)
	Awkward situations (10)
	The urine measurements and registrations were complicated (7)
Problematic to remember (53)	It is difficult to remember (20)
	The need for adult guidance (17)
	Challenges in school (16)

#### Experiences of how to manage

*This could be done in a better way*. Children described the experiences of managing technicalities of the alarm as unsatisfying. Several children wanted the alarm to be designed differently, with, for instance, bespoke signals. One suggestion for improvement, by many parents and some children, was a mobile phone app that could provide information, motivation and reminders. A few parents saw potential for improvement in the healthcare system. They expressed that the child’s constipation was discovered too late, resulting in delayed treatment of enuresis.

"…*you could have the alarm on the phone or something like that…it would be much better, I think, to avoid that super-annoying sound…you might not have…exactly the same one all the time [alarm signal], that would be really annoying" (child 8)*

"*…perhaps you can start thinking about it [constipation] earlier because they have to*…*be 6–7 years old before you can seek help [for bedwetting]*…*then you might start thinking*…*‘Well*, *could my child be constipated‴ (parent 2)*

*To be committed*. A few children explained that they did what they were supposed to do and did not miss a thing, even if they experienced that it sometimes was hard. In the beginning, they needed reminders from a parent, but after a while they managed by themselves. Parents usually stressed the importance of rigorously following the instructions given, even if this was challenging.

*“I didn’t forget at all [standard urotherapy]. I took care of myself and did everything I was supposed to do” (child 6)*

*Finding a way to remember*. Some children and parents reported strategies used in order to remember registrations in voiding charts and bladder diaries and/or standard urotherapy. The strategies were to have objects functioning as visual cues to what they were supposed to do, or to link the new procedures to already existing routines.

*"…it’s much easier when you’re at school…then you know when to do everything [standard urotherapy]" (child 6)*

*The child´s strategies*. A few children experienced that their own individual strategies made management easier. Self-made toilet and drinking schedules worked well for some children. One child explained that “you have to face the situation and just do it”, whereas some children explained that they realized that if they went to the toilet immediately when the body signaled the need for a bowel movement, they could elude the enema.

*"…go as soon as I need to…then I don’t have to take the horrible thing [enema]!" (child 9)*

*Solving problems*. Minor problems may arise during the treatment, problems that some families solved on their own. With adjustments, things worked better for the child and treatment could continue. Examples of adjustments include flavoring the medicine (PEG) with different juices or finding alternatives, such as kitchen utensils for urine measurements when not being at home.

"…*you can mix strawberry juice. And raspberry juice. And pear juice. I usually have strawberry or raspberry…those are the ones I usually have the most" (child 2)*

"…*you may have to do something even if it is…*.*weekend*…*if you are out or going away*…*then you have to remember to bring something that you can measure it in*.*" (parent 3)*

*Distractors during enema administration*. After the experience of receiving an enema, most of the children and parents found the administration troublesome, but they did also discover that the child could be encouraged by a reward or distracted with a mobile phone or tablet (an effective way to make the procedure acceptable for the child).

"…*you take your mobile phone and can play a bit while you’re at it. When using the enema. Because then you think about something else.” (child 2)*

#### Managing procedures was a favorable experience

*The urine measurements were interesting and easily registered*. The measuring of urine was easy and exciting for most of the children and parents. The majority of the parents experienced that the measurements were straightforward to perform and registration in the voiding charts with paper and pencil was considered an easy method. Most of the children observed that the amount of urine in the bladder differed from time to time, and several children tried to break a personal record regarding how much urine they could produce (which, it should be added, was not in accordance with the instructions given). A few children continued measuring urine after the three-day registration, just for fun.

"*It’s fun to measure, especially when you really need to pee…because then you know…if you’ve been holding it in for quite a while. If you have forgotten, then it is a lot. It has worked quite well to measure it and it was fun too!” (child 11)*

*Standard urotherapy was easy to manage*. Standard urotherapy was described as easy to manage and many of the children liked having a toilet schedule with stickers to mark when they had urinated. A majority of the children and parents expressed a favorable attitude towards standard urotherapy, since it coincided with already existing routines for the child.

”*Easy, I just remembered!” (child 12)*

"*I also think it has to do with the fact that it’s not strange for you*, *because you’re really good at drinking and even going to pee*, *so it wasn’t difficult for you*.*" (parent 11)*

*Favorable experiences of constipation treatment*. Regarding the treatment of constipation, PEG was expressed as easy to drink and facilitated bowel movements for all the children. Some children knew that the enema treatment was just for a limited time. As the constipation treatment continued, some children felt better and understood that the enemas were good for them. Some parents experienced an improvement in the child’s well-being after the start of enema treatment; the child felt better, had less fecal incontinence and better appetite.

"*I sat all evening in the bathroom and then I really pooped…it felt nice because sometimes I’d kept it [poop] in for a whole day." (child 11)*

*Gratitude for increased knowledge*. Some parents explained that when they initially received basic information about the child’s condition and treatment, they became calmer and gained a more confident attitude towards the treatment. For example, the knowledge about why the enemas were needed made them better motivated to carry out the recommended procedures at home.

"*…didn’t really know how this worked, why you wet yourself…so it’s been interesting to learn…I hadn’t thought about him being constipated because he usually went to the toilet anyway…and then you thought ‘yes, but what a good thing, he’s been to the toilet’ like…they start to wipe themselves and so on. Yes. It was, interesting…I think a lot of people don’t know that" (parent 2)*

*Alarm treatment being satisfying*. Some children and parents were satisfied with the enuresis alarm. They expressed that the treatment worked well and resulted in dry nights. Even though children found the treatment somewhat difficult in the beginning, they soon were used to it and thought it was worth the effort to continue.

*"…I feel satisfied with the treatment…it was at the beginning. Then you were very tired and you usually slept very deeply and all night.” (child 5)*

#### Managing procedures made it complicated

*Complications related to alarm treatment*. Many children experienced the enuresis alarm as troublesome since it was difficult for them to wake up at night, and when they did wake up they were confused and could neither turn off the device nor understand the need to go to the toilet. The morning after, they remembered nothing. The alarm sensor in the underwear was sometimes described as uncomfortable and difficult to attach. A couple of children became afraid of the alarm and thereafter had difficulties falling asleep in the evenings. For several families, alarm treatment was aggravated by excessive sensitivity of the sensor, causing false alarms.

*"That one was difficult too…sometimes it beeped when I was sleeping even though there hadn’t been any pee in the bed, so it beeped anyway and it was a real nuisance. That was what was difficult, it only went off sometimes [the alarm] and then it was very difficult…I went to the bathroom. Yes. Although I hadn’t peed in the bed." (child 14)*

*Standard urotherapy was difficult to manage*. Many children expressed that the standard urotherapy was difficult to manage and some stated that eight weeks was too long. Some children considered it difficult to drink without being thirsty and some children said that the scheduled toilet visits interrupted their activities. A few children liked the toilet diary with stickers but this was only motivating and fun in the beginning. Several children explained that they did not use the footstool (provided to optimize toilet position) since they did not see any benefit and associated it with babies.

*"This one wasn’t very helpful…It feels like when I was so sad I couldn’t reach the toilet lid; I used one just like this to reach up, so this feels like my stool when I was a baby … I can do very well without this” (child 9)*

*Constipation treatment was troublesome*. Constipation treatment (PEG and enemas) was difficult to manage and a few children made the reflection that it helped for bowel movements but not the bedwetting. The citrus fruit taste of the PEG was hard to accept for some children but the enema was described as even worse for some children. It was difficult for most of the children to relax during the rectal administration of the enemas and the emptying phase was usually described as uncomfortable, or even painful. A majority of the parents expressed that they suffered with their child. Several children experienced the enema treatment so unpleasant that they never wanted to go through it again.

*“This is the worst I have ever gotten! I wouldn’t want that again…I never want [the enema] again! “(child 9)*

*Awkward situations*. Treatment of enuresis entails the use of specific objects such as measure-cups for urine, alarm devices and water bottles. Children described these objects as posing a risk of being revealed as a bedwetter, if friends visit and see them. The water bottle was described as bothersome when no one else in school uses one. Children expressed that they were embarrassed and that they needed to come up with strategies to explain why they have an object associated to enuresis or constipation treatment.

"*…the night I peed [for the registration]…I had friends at home…I didn’t want to say that I measure my pee. So I lied to them a bit then. For example, ‘I have to make a list for something.’ So, I didn’t say ’I measure my pee because I pee in my bed’." (child 8)*

*The urine measurements and registrations were complicated*. A few children found the urine measurements troublesome because they wanted to do something fun instead, or they considered them unpleasant to handle. A minority of the parents explained that if urine measurements were scheduled during a weekend and something unplanned happened, everything had to be done all over again, another weekend.

“*It was that thing that you had to put in the toilet, I thought it was quite a pain. Because every time you had to pee, you had to take it and sometimes I would pee in the other toilet…when you watch a movie, you don’t want to miss anything" (child 8)*

“*… and it was a bit difficult*. *We started the routine*, *and thought ‘now we’ll do it this weekend’ and then it turned out that we went to some friends and then we got out of sync a bit and had to change and do it all again the next weekend and so*. *So*, *it was mostly because it was quite technical*, *to remember and when there is a panic ‘I have to go and pee’ and then you have to run first and get the measuring thing and then*.*" (parent 3)*

#### Problematic to remember

*It is difficult to remember*. After having experienced standard urotherapy most of the parents expressed that it was difficult, even for them, to remember such things as registration, regular water intake and toilet routines. Some of the children and parents explained that in the beginning everything went well, but as everyday life went on, they forgot more and more about the instructions. Some children explained that they have other things, such as play, on their minds. A few parents expressed that since they did not manage to remind the child as they should the child did not comply with the treatment.

"*At first you remember very well and then in the end, you think about something else and then you forget a little." (child 12)*

"*I also think that it has been difficult to keep to these times [urotherapy]*…*I have now been the one who has been bad at maybe reminding at the right time" (parent 6)*

*The need for adult guidance*. Both children and adults were aware of the child’s need of being reminded by parents. Children expressed that it was difficult if they were not reminded and, therefore, did not comply with the treatment.

"…*sometimes I forgot to take it [PEG] and once at my father’s place, I forgot to take it…because he didn’t put it on the table…[at my mother’s] then it was in the bag, so sometimes I forget about it if she doesn’t remind me.” (child 11)*

*Challenges at school*. During standard urotherapy in particular, most children divulged several difficulties in school. They described that even though they tried hard, it was problematic to adhere to new routines such as regular toilet visits and water intake during school hours. Some children were not allowed to go to the toilet during lessons and many children were not allowed to bring the water bottle into the classroom. The children could not rely on an adult at school to help them to remember the new routines and that made it difficult to follow the instructions. Mostly, parents were aware of the complicated situation in school and due to their limited involvement they had to rely on the child’s resources.

"*Parent: … we had written to the teacher so she knew, twice, or maybe three times.*

Child: But she didn’t remind me.

*Parent*: *No*, *I think you’ve had to remember a lot yourself; you’ve done this very well; you’ve tried to remember yourself to go to the bathroom*.*” (child and parent 12)*

## Discussion

This study is unique as it describes children´s and parents’ experiences of management and treatment of enuresis with or without concomitant constipation. There is a lack of studies with similar content; therefore, only partial comparisons can be made. Findings from the study were mixed. On one hand, families reported favorable experiences and strategies to comply with the instructions given. From this, we can learn how to better empower the families before and during treatment of enuresis. On the other hand, we found that management of enuresis treatment can imply troublesome situations and be challenging for the families, with new routines and difficulties to combine them with normal daily life. This is important to know since the medical and behavioral treatment of enuresis, with or without concomitant constipation, is heavily dependent on adherence from the families.

Our study shows that completing voiding charts and bladder/bowel diaries are experienced to require time and planning, and therefore suggested to do most of the urine measurements during a weekend. Many families keep forgetting to follow the instructions and adhere to the schedules. However, to understand the child´s enuresis, voiding charts and bladder/bowel diaries are considered crucial [4]. This study can provide suggestions on how families might increase the chances of success, such as keeping the weekend clear of other tasks or using visual reminders. Linking the new routines to existing routines may also be a fruitful strategy. However, it is important to continue the research on how important these registrations are (or are not) in enuresis, in order to reduce the burden on families. If the registrations are found not to be helpful in treatment of enuresis, then they should be omitted.

Children in the study were mostly satisfied with enuresis treatment despite the fact that they did not achieve complete dryness at night [11, 22], suggesting they may value reassurance and tools to manage the condition. Furthermore, a majority of the children expressed that it is difficult to remember new routines and the parents find it difficult to remember to remind the child, resulting in lower adherence. These results are interesting, since encouragement and support from healthcare professionals have been found to increase adherence [25]. However, in another study parents said that they were too busy to help their child with the treatment [26]. More research regarding how to support parents in helping their child is needed. Maybe a mobile phone app could be useful. Children and parents suggested that the functions of a mobile phone app such as age-adapted information, preparations, treatment, reminders and instant evaluations could facilitate the management. This is supported by previous studies [27, 28].

Standard urotherapy was considered easy to manage for families who already had routines similar to the instructions given, but it was more difficult for children who forgot about it, lost their motivation, were not thirsty or just thought it interrupted their activities. It should be noted that none of the children in our present study had any daytime incontinence, and therefore the motivation for daytime routines may have been lacking. It may have been easier to be motivated to follow such routines if a clear link was seen between the daytime routines and daytime dryness. International treatment guidelines have traditionally recommended standard urotherapy as first-line treatment for enuresis, but this approach is currently reconsidered, as recent studies show no evidence to support these recommendations [11, 12]. By removing standard urotherapy as a first-line treatment of enuresis, the number of failures, before finding the right treatment for the individual child, will reasonably be reduced.

An important result is how children experience their school day, where urotherapeutic recommendations may be difficult to adhere to. Admittedly, this situation differs between countries and cultures. Prescriptions of regular fluid intake was experienced as complicated by restrictions of having a water bottle in the classroom. Moreover, if the child did not see the bottle, they forgot to drink the water. Some children explained that they could not visit the toilet during lessons since they were expected to do that during breaks, an arguably unreasonable demand since children in early school age are too occupied by play. Children and parents found it hard to get teachers to help the child with the regular toilet visits and water intake. Teachers may not have regarded this as their responsibility or may simply have been unaware of the problem. According to the results of this study, confirmed by other research [29], the school staff need to take a leading role in health promotion, including giving the opportunity for children to take care of their bowel and bladder function, a basic human need.

Some children opined that the benefits of enuresis treatment, although uncomfortable, outweighed the difficulties. However, other children and parents gave the opposite judgement. When children really disliked an experience, for example the enema, they were frank in their choice of words: “I hate it”, “I will never do it again” or “It´s the worst thing I´ve ever done!”. One boy stated that he now goes to the toilet to empty his bowel daily just to avoid the enema. This problem could be avoided by offering alternatives when disimpaction is recommended, such as high-dose oral PEG [30]. A common strategy was to distract the child by using a mobile phone or tablet during the difficult procedure. Many children with experiences of enema administration liked the distraction and felt that it helped them endure the procedure. This strategy could be recommended to children needing enemas.

The alarm is a well-established first-line treatment of enuresis. The labor-intensive nature of the method is also well-known [9]. Nevertheless, what the children themselves think about the alarm is less well studied. Our study shows that children responding to the alarm found the treatment easy to bear but when the alarm treatment is experienced as troublesome, they did not see the benefit of using it. Technical issues with false alarms caused confusion among both children and parents. Some children found the alarm signal frightening and some children explained that they did not dare to fall asleep because of this. Of course, this is not the intention of alarm treatment. This fear needs to be proactively addressed by the healthcare professional. During alarm treatment (and standard urotherapy), the nurse made regular follow-up calls. Surprisingly, only one family communicated back to the researcher about the problems leading to corrections in the technique to avoid the false alarms. Thus, this study can show that it is important for healthcare providers to be aware of these issues and actively encourage parents to communicate any difficulties they experience. In this study some children expressed that during the night when the alarm went off, they felt confused and did not understand how to turn it off or that they had to go to the toilet. The statements show that children are dependent on a parent to manage the alarm treatment and that not having this support could be a reason why treatment does not work or is prematurely terminated.

Our study reveals that the instructions and specific objects we provide to the child may jeopardize their strategies for keeping their enuresis private. To see the measuring cup or water bottle may help the child to remember using them, but may also be discovered by visiting friends. This is important to understand when developing incontinence products. In another interview, study children explained that they have to use various strategies to hide their daytime incontinence and to avoid being embarrassed in front of friends [6]. It is known that constipation and enuresis affect children’s self-esteem as well [5–7]. A boy in this study with much experience of hiding his enuresis and treatment from friends thought a mobile phone app could help reduce the stress of being discovered. Labor-intensive procedures at home, prescribed by healthcare providers deserve more attention. The results from this study can contribute to improving and developing future care for children with enuresis, constipation and daytime incontinence.

### Strengths and limitations of the work

Interviewing children is not easy but important, especially since there is a lack of studies focusing on the child’s experiences. A parent was invited with the child to the interview but it could be argued that this should not have been done. The intention was to make the child feel comfortable and confident in the interview situation. The gender of the parents might influence the children’s responses, however in a country as Sweden there is a context of high equality between mothers and fathers and therefore we do not see a risk that the child would be impacted by which parent accompanied them.

Another obstacle was the children’s age, six to seven years (n = 10), which throughout the study turned out to be the most difficult age to interview. In a recent review, the authors concluded that there is a lack of studies with children of this age participating. Ages six to eight years represent 6% of the child participation in qualitative research [31]. Therefore, this study is extra important. It should be noted that the children in the study did not have a neuropsychiatric diagnosis, which is common in children with enuresis [32]. This may be considered a limitation of the study. However, it is possible that some of the children were diagnosed at a later stage, as they were of lower school age at the time of the study and had not yet been diagnosed.

The venue could also be objected to since interviews mostly took place in a hospital setting [20]. Due to Covid restrictions, a couple of interviews (pilot study) were held online, resulting in the child getting distracted by the other parent, siblings or toys. Furthermore, Covid protocols might impact school or other routines. However, in Sweden children continued going to school as usual during the pandemic. To meet physically for the interview improved the situation but it was still difficult for children to express their experiences just by answering questions. This is the reason why children were presented with the actual objects that they knew from the procedures they had performed at home. Researchers interviewing children are recommended to use objects [23, 24, 31], but this interview technique seems not to be common in pediatric research. This experience highlights the importance of considering alternative methods when interviewing children.

There is a risk that children and parents under- or over-reported their experiences. The fact that the interviewer was the child’s healthcare provider may therefore be a drawback. On the other hand, it can be argued that since children (and parents) already had an established professional relationship with the interviewer they were more confident in sharing their thoughts. To minimize social desirability the interviewer had an empathic and non-judgmental approach. Children felt that they could honestly express how much they disliked constipation and alarm treatment, if that was the case. Children are more likely to tell the truth if they are questioned by someone they know has knowledge of their situation [33]. The total number of included children in the present study (n = 14), together with one parent, might be seen limited but analysis of data showed redundancy and thus, the sample size was large enough [20]. A qualitative exploratory study design was used to find different perspectives and try to understand the informants’ views instead of explaining results being generalizable to a wider population. However, carefully collected, analyzed and described, the findings can be transferred to similar persons or contexts.

#### Recommendations for further research

More research is needed, as there is little knowledge about how healthcare is experienced by children with enuresis, especially children in early school age. It is important that there be no large discrepancy between what the healthcare system requires and what is feasible at home, since this would increase the risk for failure. Even more important is to involve children more in future research, not only as interviewees but also as participants in the process of designing the research project in order to find out what is important for children to discover and develop.

#### Implications for policy and practice

In order to adhere to treatment of enuresis the children need support from their parents. The healthcare provider, in collaboration with the parents, needs to find options to suit the individual families. Using visual reminders or connecting tasks with already existing routines were successful strategies. A mobile app was suggested as a possible solution to facilitate treatment, e.g. for motivation, registrations, reminders, individualization of the enuresis alarm signal, and as a source of information about the child’s condition. Furthermore, one child noted that a mobile app could help with maintaining privacy as well.

Children treated with standard urotherapy are prescribed regular fluid intake and toilet visits during the day, which could be argued to be a healthy strategy for every schoolchild! Children must be taken seriously and be invited to work together with parents, school staff and healthcare providers to find ways to implement this strategy in the long term.

Enema and the enuresis alarm aroused strong feelings. Knowledge of this is important and alternative methods must be available for these children. When treated, children need to be empowered to manage their situation, like using distractors during enema administration or reporting alarm problems for support.

Objects used for treatment of enuresis can jeopardize children’s attempts to keep their condition private.

It is important to refine current drugs and products for treating enuresis and constipation or develop alternatives to optimize adherence and positive treatment result.

## Conclusion

Managing the treatment of enuresis at home can be challenging for children. There were procedures that children did not like and routines were difficult to remember, leaving children in a complicated situation. However, the study can suggest favorable strategies in how to manage and overcome these difficulties, and the increased knowledge gained provides an opportunity to empower families before and during enuresis treatment. This is important, since the treatment of enuresis is heavily dependent on adherence from the families.

## References

[1] Bower WF, Moore KH, Shepherd RB, Adams RD. The epidemiology of childhood enuresis in Australia. Br J Urol. 1996;78(4):602–6 doi: 10.1046/j.1464-410x.1996.13618.x 8944518

[2] Bakker E, van Sprundel M, van der Auwera JC, van Gool JD, Wyndaele JJ. Voiding habits and wetting in a population of 4,332 Belgian schoolchildren aged between 10 and 14 years. Scand J Urol Nephrol. 2002;36(5):354–62 doi: 10.1080/003655902320783863 12487740

[3] Cederblad M, Nevéus T, Åhman A, Österlund Efraimsson E, Sarkadi A. "Nobody asked us if we needed help"—Swedish parents’ experiences of enuresis. J Pediatr Urol. 2014;10(1):74–9 123 doi: 10.1016/j.jpurol.2013.06.006 23849996

[4] Nevéus T, Fonseca E, Franco I, Kawauchi A, Kovacevic L, Nieuwhof-Leppink A, et al. Management and treatment of nocturnal enuresis-an updated standardization document from the International Children’s Continence Society. J Pediatr Urol. 2020;16(1):10–9 doi: 10.1016/j.jpurol.2019.12.020 32278657

[5] Tai TT, Tai BT, Chang YJ, Huang KH. The Importance of Understanding Parental Perception When Treating Primary Nocturnal Enuresis: A Topic Review and an Institutional Experience. Res Rep Urol. 2021;13:679–90 doi: 10.2147/RRU.S323926 34522688 PMC8434936

[6] Malhotra NR, Kuhlthau KA, Rosoklija I, Migliozzi M, Nelson CP, Schaeffer AJ. Children’s experience with daytime and nighttime urinary incontinence—A qualitative exploration. J Pediatr Urol. 2020;16(5):535.e1-.e8 doi: 10.1016/j.jpurol.2020.10.002 33148456 PMC9764822

[7] Belsey J, Greenfield S, Candy D, Geraint M. Systematic review: impact of constipation on quality of life in adults and children. Aliment Pharmacol Ther. 2010;31(9):938–49 doi: 10.1111/j.1365-2036.2010.04273.x 20180788

[8] Lag (2018:1197) om Förenta nationernas konvention om barns rättigheter [Act on the United Nations Convention on the Rights of the Child, 2018] (2018).

[9] Nevéus T, Eggert P, Evans J, Macedo A, Rittig S, Tekgül S, et al. Evaluation and treatment of monosymptomatic enuresis—a standardisation document from the International Children’s Continence Society (ICCS). J Urol. 2010;183:441–7 10.1016/j.juro.2009.10.043 20006865

[10] Cederblad M, Engvall G, Sarkadi A, Nevéus T. No effect of basic bladder advice in enuresis–a randomised controlled trial. J Pediatr Urol. 2015;11(3):153e1–5 10.1016/j.jpurol.2013.06.006 25975733

[11] Borgström M, Bergsten A, Tunebjer M, Hedin Skogman B, Neveus T. Daytime urotherapy in nocturnal enuresis: a randomised, controlled trial. Arch Dis Child. 2022;107(6):570–4 doi: 10.1136/archdischild-2021-323488 35074830 PMC9125372

[12] Jørgensen CS, Kamperis K, Walle JV, Rittig S, Raes A, Dossche L. The efficacy of standard urotherapy in the treatment of nocturnal enuresis in children: A systematic review. J Pediatr Urol. 2022 doi: 10.1016/j.jpurol.2022.12.011 36641240

[13] Neveus T, Lackgren G, Tuvemo T, Hetta J, Hjalmas K, Stenberg A. Enuresis—background and treatment. Scand J Urol Nephrol Suppl. 2000(206):1–44 11196246

[14] Loening-Baucke V. Prevalence rates for constipation and faecal and urinary incontinence. Arch Dis Child. 2007;92(6):486–9 doi: 10.1136/adc.2006.098335 16857698 PMC2066162

[15] McGrath KH, Caldwell PHY, Jones MP. The frequency of constipation in children with nocturnal enuresis: a comparison with parental reporting. J Paediatr Child Health. 2008;44(1–2):19–27 doi: 10.1111/j.1440-1754.2007.01207.x 17854414

[16] Mugie SM, Benninga MA, Di Lorenzo C. Epidemiology of constipation in children and adults: a systematic review. Best Pract Res Clin Gastroenterol. 2011;25(1):3–18 doi: 10.1016/j.bpg.2010.12.010 21382575

[17] Kim JH, Lee JH, Jung AY, Lee JW. The prevalence and therapeutic effect of constipation in pediatric overactive bladder. Int Neurourol J. 2011;15(4):206–10 doi: 10.5213/inj.2011.15.4.206 22259734 PMC3256305

[18] Hunsballe JM, Hansen TK, Rittig S, Nørgaard JP, Pedersen EB, Djurhuus JC. Polyuric and non-polyuric bedwetting–pathogenetic differences in nocturnal enuresis. Scand J Urol Nephrol. 1995;S173:77–9 8719573

[19] Chase J, Austin P, Hoebeke P, McKenna P. The management of dysfunctional voiding in children: a report from the Standardisation Committee of the International Children’s Continence Society. J Urol. 2010;183(4):1296–302 doi: 10.1016/j.juro.2009.12.059 20171678

[20] Patton MQ. Qualitative Research & Evaluation Methods. Fourth ed. London: SAGE Publications Ltd; 2015.

[21] Silverman D. Interpreting Qualitative Data. 6 ed: Sage Publications Ltd.; 2020 2020.

[22] Borgström M, Bergsten A, Tunebjer M, Hedin Skogman B, Nevéus T. Fecal disimpaction in children with enuresis and constipation does not make them dry at night. J Pediatr Urol. 2022;18(4):446.e1-.e7 10.1016/j.jpurol.2022.05.008 35718673

[23] Salmon K. Remembering and reporting by children: The influence of cues and props. Clin Psychol Rev. 2001;21(2):267–300 doi: 10.1016/s0272-7358(99)00048-3 11293368

[24] Salmon K, Pipe M-E. Recalling an event one year later: the impact of props, drawing and a prior interview. Appl Cogn Psychol. 2000;14(2):99–120 10.1002/(SICI)1099-0720(200003/04)14:2<99::AID-ACP639>3.0.CO;2-5

[25] Larsson J, Borgström M, Karanikas B, Nevéus T. Can enuresis alarm therapy be managed by the families without the support of a nurse? A prospective study of a real-world sample. Acta Paediatr. 2023;112(3):537–42 doi: 10.1111/apa.16634 36527281 PMC10107766

[26] Morison MJ, Tappin D, Staines H. ’You feel helpless, that’s exactly it’: parents’ and young people’s control beliefs about bed-wetting and the implications for practice. J Adv Nurs. 2000;31(5):1216–27 doi: 10.1046/j.1365-2648.2000.01426.x 10840256

[27] Whale K, Beasant L, Wright AJ, Yardley L, Wallace LM, Moody L, et al. A Smartphone App for Supporting the Self-management of Daytime Urinary Incontinence in Adolescents: Development and Formative Evaluation Study of URApp. JMIR Pediatr Parent. 2021;4(4):e26212 doi: 10.2196/26212 34779780 PMC8663506

[28] Bernard S, Boucher S, McLean L, Moffet H. Mobile technologies for the conservative self-management of urinary incontinence: a systematic scoping review. Int Urogynecol J. 2020;31(6):1163–74 doi: 10.1007/s00192-019-04012-w 31267139

[29] Michels N, Van den Bussche K, Vande Walle J, De Henauw S. School Policy on Drinking and Toilets: Weaknesses and Relation With Children’s Hydration Status. J Nutr Educ Behav. 2019;51(1):32–40 doi: 10.1016/j.jneb.2018.07.001 30146453

[30] Vriesman MH, Koppen IJN, Camilleri M, Di Lorenzo C, Benninga MA. Management of functional constipation in children and adults. Nat Rev Gastroenterol Hepatol. 2020;17(1):21–39 doi: 10.1038/s41575-019-0222-y 31690829

[31] Hunleth JM, Spray JS, Meehan C, Lang CW, Njelesani J. What is the state of children’s participation in qualitative research on health interventions?: a scoping study. BMC Pediatr. 2022;22(1):328 doi: 10.1186/s12887-022-03391-2 35659206 PMC9166159

[32] de Sena Oliveira AC, Athanasio BDS, Mrad FCC, Vasconcelos MMA, Albuquerque MR, Miranda DM, et al. Attention deficit and hyperactivity disorder and nocturnal enuresis co-occurrence in the pediatric population: a systematic review and meta-analysis. Pediatr Nephrol. 2021;36(11):3547–59 doi: 10.1007/s00467-021-05083-y 34009466

[33] Hartwig J, Wilson JC. Factors affecting children’s disclosure of secrets in an investigatory interview. Child Abus Rev. 2002;11(2):77–93 10.1002/car.725

